# The relationship between implicit absenteeism and grit and compassion fatigue in female neonatal nurses

**DOI:** 10.3389/fpubh.2025.1530584

**Published:** 2025-04-30

**Authors:** Xushu Chen, Xiaowen Li, Shulin Hou, Ru Yang, Zeyao Shi

**Affiliations:** ^1^Department of Neonatology Nursing, West China Second University Hospital, Sichuan University, Chengdu, China; ^2^Key Laboratory of Birth Defects and Related Diseases of Women and Children (Sichuan University), Ministry of Education, Chengdu, Sichuan, China

**Keywords:** implicit absenteeism, influencing factors, compassion fatigue, grit, neonatal, nurses

## Abstract

**Introduction:**

Implicit absenteeism is a growing concern among nurses, as it may reduce nursing productivity and affect the quality of nursing services and patients’ health. This study aimed to investigate the status and influencing factors of implicit absenteeism among female nurses in neonatal units and to explore the correlations between compassion fatigue, grit, and implicit absenteeism.

**Method:**

An anonymous online questionnaire was administered to female nurses in a neonatal unit from August to October 2024 using the General Information Questionnaire, Stanford Implicit Absenteeism Scale, Compassion Fatigue Short Scale, and self-reported short Grit Scale (Grit-S).

**Results:**

In total, 269 female neonatal unit nurses were included in our study. The implicit absenteeism score of female neonatal nurses was (14.41 ± 4.08). Age, education, and years of work experience were the main factors influencing implicit absenteeism among female nurses in the neonatal unit (all *p* < 0.05). In addition, implicit absenteeism was positively correlated with compassion fatigue (*r* = 0.672, *p* < 0.01) and grit (*r* = 0.420, *p* < 0.01). Additionally, compassion fatigue was positively correlated with grit (*r* = 0.559, *p* < 0.01).

**Conclusion:**

Female nurses in neonatal units have high rates of implicit absenteeism. Clinical managers should pay particular attention to the implicit absenteeism and physical and mental health of female nurses with bachelor’s degrees or above, those over 30 years of age, and those with more than 10 years of work experience. Simultaneously, governments and healthcare organizations need to develop interventions to promote the physical and mental health of nurses, reduce implicit absenteeism, and further contribute to the stable development of the nursing workforce.

## 1 Introduction

Neonatal nurses play a crucial role in reducing neonatal mortality and improving the quality of child survival (1). The World Health Organization has shown that higher numbers of female nurses and health service coverage are negatively associated with infant mortality (2). However, owing to the complexity and specialized nature of neonatal care, they can pose multiple stressors and challenges. According to the Cognitive Activation Theory of Stress (CATS) (3), prolonged and excessive stress may be detrimental to individuals (4). One of the critical issues to consider is the implicit absenteeism of nurses (coming to work when sick), which may seriously affect the quality of care and neonatal safety (5).

Implicit absenteeism is defined as when an employee is physically present at work despite experiencing physical or mental health problems (6). This results in unsatisfactory work performance, despite apparent attendance. Previous studies (7) have shown that implicit absenteeism among nurses is strongly associated with falls, medication errors, and lower quality of care. It may also trigger a deterioration in nurses’ health status, leading to additional negative impacts, such as impaired health productivity and economic losses (8). A cross-sectional study by Dhaini et al. (9) on absenteeism and implicit absenteeism and their relationship with the psychosocial work environment among 3,176 caregivers in Switzerland found that the rates of absenteeism and detection of implicit absenteeism among caregivers were 15.6 and 32.9%, respectively. In China, implicit absenteeism is common among intensive care unit nurses (8). Nurses may refuse to take time off due to a “sense of duty,” but prolonged resource overdrafts can exacerbate implicit absenteeism (10), as grit may evolve into “excessive persistence” (11).

Grit is an essential psychological quality (12) that plays a crucial role in nurses’ job competence, job satisfaction, and overall performance (13). This is because it increases nurses’ willingness to return to work, thereby reducing turnover and increasing retention (14). A study of 514 registered nurses in Finland reported moderately high grit levels (mean = 3.82, SD = 0.44) (15). Another study of 231 nurses from 11 blood purification centers in China found moderate levels of nurse grit (16). The Job Demands-Resources (JD-R) model proposed by Bakker and Demerouti at the turn of the century (17) states that job resources, including grit, motivate individuals to create positive work outcomes and reduce mental health problems and burnout. However, excessive levels of grit seem to cause nurses who are instantly physically exhausted and stressed to continue to work despite the frustrations and difficulties they may encounter in the work environment (14). However, this seems to have significantly increased hidden absenteeism among nurses. Currently, no studies are available on grit among neonatal intensive care unit (NICU) nurses. The relationship between perseverance and hidden absenteeism remains poorly understood and requires further study.

For neonatal nurses, close contact with babies and providing comprehensive physical, psychological, social, and cultural care to them and their families require significant physical and emotional commitment (18). It is common knowledge that compassion occurs when nurses are emotionally drained from empathy while helping. Compassion can help nurses put themselves in the patients’ shoes, better understand patients’ emotions, and alleviate their suffering (19). However, nurses are chronically exposed to resuscitation situations and trauma in children (4). Compassion fatigue occurs when the total amount of compassion exceeds the nurse’s ability to recover and cope (20). The JD-R model states that high workloads and stress can lead to individuals experiencing burnout at work, impairing their physical health and causing them to attempt to maintain their job performance through implicit absenteeism. This may further increase implicit absenteeism among nurses (21). A study from Australia suggested that improving compassion fatigue in emergency department nurses might help limit nurse burnout, a concept closely related to implicit absenteeism (22). Therefore, as researchers increasingly recognize the value of clinical nurses’ mental health, exploring the possibility of empathy fatigue is crucial for understanding their implicit absences.

However, based on the above literature review, we found little research on implicit absenteeism among neonatal nurses. Therefore, using the JD-R as our theoretical framework, this study aimed to explore the relationship between implicit absenteeism, grit, and compassion fatigue among female nurses in a neonatal unit. This may provide important insights for interventions aimed at improving implicit absenteeism among nurses. The key terms used in this study are listed in Table 1.

**Table 1:** Descriptors for key terms.

Key term	Description
Implicit absenteeism	The phenomenon of employees who should have taken sick leave when they are in poor physical or mental health, but insist on going to work and attending to their illness, with a consequent loss of health productivity (7).
Grit	Passion and persistence for long-term goals (12).
Compassion Fatigue	A combination of physical, emotional, and spiritual depletion associated with caring for patients in significant emotional pain and physical distress (59).

## 2 Methods

### 2.1 Participants and study design

This study was conducted between August and October 2024 using a purposive sampling method from a tertiary-level maternal and child healthcare hospital with 307 neonatal beds in Chengdu, Sichuan Province, China. The inclusion criteria for the study population were (1) female nurses in the neonatal unit, (2) working for >1 year, and (3) voluntary participation in the survey. The exclusion criteria were: (1) nurses undergoing training, refresher training, and rotation and (2) registered nurses, including specialists and practice nurses, who were outside the hospital where they worked.

Our study was approved by the Research Ethics Committee of West China Second Hospital of Sichuan University before the study started. Invitations to participate were sent to all eligible nurses via the Questionnaire Star App; 20 of them declined to participate. The remaining 280 eligible nurses scanned the QR codes to complete the questionnaire. The study was conducted in an anonymous response format. The final results were imported from Questionnaire Star into Excel, and data validation was performed using another quality controller.

### 2.2 Measurements

#### 2.2.1 Basic sociodemographic data

This study used a self-administered demographic questionnaire to gather information on participants’ characteristics, including age, title, education, marital status, years of work experience, and child rearing.

#### 2.2.2 Implicit absenteeism

The Stanford Implicit Absenteeism Scale was developed by the Stanford University (23). This scale was developed by Fang et al. (24). The Cronbach’s *α* of the Chinese version of the scale ranged from 0.76 to 0.90, with good reliability and validity. Previous research (25) has shown that this scale can be used to measure implicit absenteeism in clinical nurses. The scale consists of 6 items and is scored on a 5-point Likert scale, with “completely disagree” scoring 1, “relatively disagree” scoring 2, “unsure” scoring 3, “relatively agree” scoring 4, “completely agree” scoring 5. The total score for each of the six items ranges from 6 to 30, with higher scores indicating more severe implicit absenteeism. The Cronbach’s *α* in this study was 0.802.

#### 2.2.3 Compassion fatigue

The Compassion Fatigue Short Scale, which was retooled for burnout by Sun et al. (26), was originally developed by Adams et al. (27). The scale consists of 13 items rated on a 10-point Likert scale. The compassion fatigue scale is scored out of a possible 130 points, with a higher total score indicating a more severe degree of compassion fatigue. Based on the average scores of the items, the degree of compassion fatigue can be classified into three categories: mild (<4 points), moderate (4–7 points), and severe (>7 points) (28). In addition, validated factor analyses indicated that the questionnaire had good construct validity (27). The Cronbach’s *α* in our study was 0.929.

#### 2.2.4 Grit

The self-reported short grit scale (Grit-S) was developed by Duckworth et al. (29) and consists of two dimensions: consistency of interest and persistence of effort. The scale consists of eight items scored on a 5-point Likert scale (1 = not at all, 5 = fully), with higher scores indicating more exceptional grit on the part of the individual. The Chinese version of the Grit-S has good reliability and has been validated by Li et al. (30). Grit is the quality of will in which people consciously overcome difficulties and strive to achieve predetermined goals. This questionnaire was used in previous studies to assess endurance and persistence (31). This scale has good reliability and validity.

### 2.3 Statistical analysis

IBM SPSS Statistics version 26.0 (Armonk, NK, United States) was used for the statistical analysis. Includes descriptive statistics to examine frequencies and percentages, and inferential statistics to examine associations between variables. One-way analysis was performed using *t*-test and ANOVA. The implicit absenteeism, compassion fatigue, and grit scores did not follow a normal distribution. Therefore, Spearman’s correlation analyses were used to examine the correlations between the scale scores. Harman’s one-way method was used to test for common method bias. A multiple linear regression analysis was used to explore the factors influencing implicit absenteeism. Variables that were statistically significant on one-way analysis were included as independent variables, and implicit absenteeism as the dependent variable. The input method was used with *α*_in_ = 0.05 and α_out_ = 0.10, and *p* < 0.05 was considered statistically significant.

## 3 Results

### 3.1 Common method bias test

In our study, an exploratory factor analysis was performed on all variables, and six factors with eigenvalues >1 were extracted. The first factor explained 10.556% of the total variance, which was below the critical value of 40% (32). Thus, we conclude that there was no significant methodological bias in this study.

### 3.2 Demographic characteristics

A total of 280 questionnaires were distributed and 269 valid questionnaires were collected, with a recovery rate of 96.1%. Table 2 shows the demographic characteristics of the female nurses in the neonatal unit. Most were under 30 years of age (56.5%) and had the title of senior nurse or above (50.2%). Regarding educational background, female nurses in the neonatal unit predominantly had a bachelor’s degree or higher (84.4%). Additionally, 191 (71.0%) nurses were married, 146 (54.3%) had worked for 10 years or less, and more than half (62.8%) were raising children. In addition, the difference in compassion fatigue among female nurses in the neonatal unit was statistically significant with respect to age, education, and years of work experience (*p* < 0.05). In contrast, the difference was not statistically significant with regard to job title, marital status, and rearing children (*p* > 0.05).

**Table 2:** Demographic characteristics of the nurses with implicit absenteeism (*n* = 269).

Characteristic	All participants *n* (%)	Score	t/F	*p*
Age			−10.618	**0.016**
≤30	152 (56.5)	12.47 ± 3.679		
>30	117 (43.5)	16.94 ± 3.063		
Job title				
Nurse	28 (10.4)	15.36 ± 3.313	1.364	0.257
Primary nurse	106 (39.4)	14.00 ± 4.204		
Senior nurse and above	135 (50.2)	14.54 ± 4.105		
Education				
Below bachelor’s degree	42 (15.6)	8.19 ± 1.756	−14.265	**<0.001**
Bachelor’s degree or above	227 (84.4)	15.56 ± 3.260		
Marital status				
Single	76 (28.3)	14.92 ± 3.922	1.122	0.327
Married	191 (71.0)	14.24 ± 4.113		
others	2 (0.7)	12.00 ± 7.071		
Working experience (in years)			−10.316	**<0.001**
≤10 years	146 (54.3)	12.41 ± 3.350		
>10 years	123 (45.7)	16.79 ± 3.563		
Raising children			−1.085	0.279
Yes	169 (62.8)	14.21 ± 4.129		
No	100 (37.2)	14.76 ± 3.985		

Bold values: *p* < 0.05.

### 3.3 Compassion fatigue, grit, and the status of implicit absenteeism

Table 3 presents the scores for each implicit entry. Q6 yielded the highest scores. Table 4 shows that the implicit absence score of female nurses in the neonatal unit was (14.41 ± 4.08). Previous studies (8) stated that the median of nurses’ implicit absence scores was the cut-off point between high and low implicit absence scores. In this study, the median implicit absence score for female nurses was 14. Therefore, 161 (59.9%) and 108 (40.1%) female nurses had high implicit absenteeism scores. In addition, the compassion fatigue score was (3.21 ± 1.61), indicating that the compassion fatigue of female nurses was at a low level. The grit score was (20.87 ± 4.32).

**Table 3:** Means and standard deviations for each entry for implicit absenteeism.

Items	Min	Max	Mean ± SD
Q1. In the past month, due to health problems, my work pressure has become more difficult to adjust.	1	5	2.46 ± 1.03
Q2. In the past month, due to health problems, I was unable to complete the difficult tasks at work.	1	4	2.15 ± 0.88
Q3. In the past month, due to health problems, I could not get pleasure from my work.	1	5	2.32 ± 1.02
Q4. In the past month, due to health problems, I felt it was impossible to carry out some work tasks.	1	5	2.15 ± 0.90
Q5. In the past month, despite my health problems, I was able to concentrate on finishing my work.*	1	5	2.59 ± 1.01
Q6. In the past month, despite my health problems, I still feel energetic and can finish all my work.*	1	5	2.73 ± 0.93

* The item was inverse-coded.

**Table 4:** Correlation between variables.

Variables	Mean	SD	1	2	3
Compassion fatigue	3.21	1.61	1		
Grit	20.87	4.32	0.559**	1	
Implicit absenteeism	14.41	4.08	0.672**	0.420*	1

*: *p* < 0.05; **: *p* < 0.01.

### 3.4 Correlation between the main study variables

In this study, implicit absenteeism was positively correlated with compassion fatigue (*r* = 0.672, *p* < 0.01) and grit (*r* = 0.420, *p* < 0.01), while compassion fatigue was positively correlated with grit (*r* = 0.559, *p* < 0.01) (see Table 4).

### 3.5 Regression analysis of implicit absenteeism

In our study, the variance inflation factor (VIF) values were all less than 10, indicating reliable linear regression multiple covariance test results. First, the variables that were significant in the univariate analysis, compassion fatigue, and grit were included in the equation as independent variables. Second, implicit absenteeism is included in the regression equation as a dependent variable. Finally, the results of multiple linear regression analysis showed that age, education, years of work experience, compassion fatigue, and grit were the main factors influencing implicit absenteeism among neonatal nurses. This equation explains 72.0% of the variance in implicit absenteeism among female nurses in neonatal units. The results are summarized in Table 5.

**Table 5:** Regression analysis of implicit absenteeism.

Variable	*b*	*se*	*β*	*t*	*p*	95%CI	VIF
Constant	−3.461	0.917		−3.775	<0.001	[−5.267, −1.656]	
Age	1.099	0.32	0.134	3.437	0.001	[0.469, 1.728]	1.452
Education	4.712	0.402	0.42	11.724	<0.001	[3.921, 5.504]	1.231
Working experience	1.94	0.309	0.237	6.286	<0.001	[1.332, 2.547]	1.366
Compassion fatigue	0.059	0.008	0.305	7.232	<0.001	[0.043, 0.075]	1.703
Grit	0.111	0.036	0.117	3.037	0.003	[0.039, 0.182]	1.422

R = 0.852, *R*^2^ = 0.725, adjusted *R*^2^ = 0.720, F = 138.913, *p* < 0.001.

## 4 Discussion

To the best of our knowledge, this is the first study to comprehensively examine the relationships among grit, compassion fatigue, and implicit absenteeism among nurses in a neonatal unit. We investigated the current status of implicit absenteeism among female nurses in a neonatal unit in Chengdu, Sichuan, China. Our cross-sectional study revealed that age, education, and work experience were influential factors in compassion fatigue. Contributing to the existing literature, we found a significant association between compassion fatigue, grit, and implicit absenteeism among female neonatal nurses. These findings are relevant to healthcare providers, policymakers, and public health practitioners as they inform potential interventions. These strategies are expected to improve implicit absenteeism and the quality of care in this population.

The results showed that the implicit absenteeism scores of neonatal nurses were severe. This was similar to the results of a cross-sectional study conducted by Lin et al. (8) with 517 intensive care unit nurses from 10 tertiary hospitals in Sichuan Province, China. Female nurses in neonatal units face various stressors including heavy workloads, missed care, and failure to resuscitate neonates, which can lead to emotional exhaustion, burnout, and implicit absenteeism (33). The frequency of such negative experiences is higher, and the psychological impact is more severe among NICU healthcare workers than those in other departments (34). The compassion fatigue scores of female nurses in the neonatal unit were lower than those reported in previous studies (28). The level of economic development and availability of health services may vary between regions (35). In addition, the grit score in our study was lower than in a survey conducted by Yang et al. (36) with 709 psychiatric nurses in southwestern China. In general, men have higher grit and resilience in the face of adversity than women (37). Thus, the difference in sex of the included patients further contributed to the better results of Yang’s study than ours.

It is worth noting that age, educational background, and years of work experience were influential factors for implicit absenteeism. Female neonatal unit nurses aged > 30 years had higher implicit absence scores than female nurses aged < 30 years, similar to the findings of previous studies (38). Most nurses over 30 years of age are the core strengths of the department, with heavy clinical tasks and teaching duties. Furthermore, they face significant pressure to promote their titles (39). Therefore, they insist on going to work even when not feeling well. Female nurses with bachelor’s degrees or higher had higher implicit absenteeism than those with bachelor’s degrees alone. Nurses with bachelor’s degrees or higher must undertake clinical and research work (40). Once sick leave is taken, the workload increases. Female nurses who have been working for more than 10 years must undertake clinical duties and participate in the quality and safety management of wards (41). Their heavy workload makes them vulnerable to physical problems and exacerbates implicit absenteeism.

This is consistent with our suggestion that grit is positively associated with implicit absenteeism, suggesting that the higher the nurses’ grit, the more severe their implicit absenteeism, which is similar to the results of previous studies (42, 43). Female nurses in neonatal units with high levels of grit treat work stress as a challenge to overcome (44). They are also willing to adopt positive coping styles and dedicate more time and resources to solving problems encountered in clinical nursing (45). As a result, when they are unwell or have health problems, they still show grit and insist on working with their illnesses, which, in turn, increases implicit absenteeism. Because of the above findings, healthcare organizations need to intervene by organizing regular physical check-ups for nurses and early identification of nurses’ physical conditions (46, 47). Moreover, managers should ensure that nurses with poor physical and mental health rest by coordinating their alternative work with other nurses. Nurses can also self-motivate, self-adjust, and take the initiative to confide, communicate, and share information through lectures by psychologists (48).

Additionally, compassion fatigue was positively associated with implicit absenteeism. Similar to the results of previous studies (49, 50). One study indicated that nurses feel physically and emotionally drained because they are unable to provide the care they desire (48). When nurses are unable to save a neonate’s life or relieve pain despite doing their best, they may feel that the value of their work has not been reflected. They may also experience physical and emotional exhaustion (51). Over time, the high volume of work performed by nurses may lead to compassion fatigue. Nurses do not have the time or energy to channel the negative effects of patient trauma, further compromising their physical and mental health (52), which, in turn, increases the likelihood of implicit absenteeism. There is a direct cost to healthcare organizations when staff begin to show signs of burnout and fatigue (53). Therefore, on the one hand, nursing managers can further improve the individual’s sense of achievement and resilience to reduce frustration by upgrading nurses’ clinical workability and professional knowledge (54). In contrast, nurse managers can receive professional psychological support through mental toughness training or stress management programs (55, 56).

These findings are consistent with the growing recognition that the focus should shift to the physical, mental, and spiritual health of healthcare workers to improve their quality of care (34). Nursing managers should focus on the mental health of nurses in neonatal units (57). At the same time, hospitals should strengthen the prevention and control of implicit absenteeism among nurses, and teams should be established to prevent and control implicit absenteeism among nurses. In addition, the government should further strengthen the construction of laws related to nurses’ occupational health in China and push forward legislative work on the Nurses Act. The physical and mental health rights and interests that nurses should enjoy also need to be highlighted. Finally, implicit absenteeism due to excessive insistence on working with illness and the occurrence of various risks and hazards can be reduced (58).

## 5 Conclusion

Female neonatal nurses in Chengdu, Sichuan, China have a high rate of implicit absenteeism. Clinical managers should pay particular attention to the implicit absenteeism status and the physical and mental health of female nurses with bachelor’s degrees or above, those over 30 years of age, and those over 10 years of work experience. Additionally, implicit absenteeism among female nurses in neonatal units is associated with compassion fatigue and grit. Nursing managers should focus on nurses with high levels of compassion fatigue and grit. Hospitals and related departments should build organizational support environments that focus on nurses’ health management practices. Simultaneously, the government should accelerate legislation to improve implicit absenteeism.

## 6 Limitations

The limitations of this study are: 1. This is a cross-sectional study. Future longitudinal studies should explore the dynamic trajectory of implicit absenteeism over time and provide more information for further research on implicit absenteeism among neonatal nurses. 2. We used self-report and retrospective scales, which may have caused reporting bias. 3. This study only surveyed neonatal nurses in a tertiary hospital in Chengdu City, Sichuan Province, which may affect the representativeness of the population and the extrapolation of the findings; a multicenter large-sample study could be conducted in the future. 4. Our study only considered factors such as age, education, and length of service and did not control for other confounding variables (e.g., workplace environment).

## Data Availability

The datasets presented in this study can be found in online repositories. The names of the repository/repositories and accession number(s) can be found in the article/supplementary material.
